# HbA1c of 6.5% to Diagnose Diabetes Mellitus — Does It Work for Us? — The Bellville South Africa Study

**DOI:** 10.1371/journal.pone.0022558

**Published:** 2011-08-12

**Authors:** Annalise E. Zemlin, Tandi E. Matsha, Mogamat S. Hassan, Rajiv T. Erasmus

**Affiliations:** 1 Division of Chemical Pathology, Faculty of Health Sciences, National Health Laboratory Service and University of Stellenbosch, Cape Town, South Africa; 2 Department of Biomedical Sciences, Faculty of Health and Wellness Science, Cape Peninsula University of Technology, Cape Town, South Africa; 3 Department of Nursing and Radiography, Faculty of Health and Wellness Science, Cape Peninsula University of Technology, Cape Town, South Africa; University of Las Palmas de Gran Canaria, Spain

## Abstract

**Background:**

HbA1c has been the gold standard for glycaemic control follow-up for decades. In 2009, a level of 6.5% (48 mmol/mol) was proposed as diagnostic for diabetes. We test this cut-off in our community.

**Methods:**

Participants (946) from a community-based study were screened for diabetes using either a fasting blood glucose or oral glucose tolerance test (OFTT). The HbA1c cut-off of 6.5% was tested for each group. A receiver operator characteristic (ROC) curve for both groups was generated to establish an optimal cut-off.

**Results:**

Our study included 224 (23.7%) males and 722 (76.3%) females. Using fasting blood glucose alone, 117 (14%) were diagnosed with diabetes −50% had an HbA1c value of ≥6.5% (48 mmol/mol). Using an OGTT, 147 (18%) were diagnosed with diabetes −46% had an HbA1c value of ≥6.5% (48 mmol/mol). ROC curves found a level of 6.1% (43 mmol/mol) to be optimal in both groups (AUC 0.85 and 0.82 respectively). The sensitivities were 80% and 75% and the specificities 77% and 78% respectively.

**Conclusions:**

A cut off of 6.5% (48 mmol/mol) is a good diagnostic tool with its high specificity; however the low sensitivity limits its use. We found a level of 6.1% (43 mmol/mol) to be optimal. This emphasizes the need for evidenced based values to be established in various population groups.

## Introduction

Diabetes is a disease fuelled by the increasing worldwide obesity epidemic with significant morbidity and mortality, and the World Health Organization (WHO) estimates that it will affect 366 million individuals worldwide by 2030 [1]. Its diagnosis was previously made either according to the WHO criteria which were updated in 2006 [2] using a fasting blood glucose sample and subsequent 75 g oral glucose tolerance test (OGTT) with blood taken for glucose determination again 2 hours after an oral glucose challenge, or according to the American Diabetes Association (ADA) criteria which were updated in 2005 [3], using only a fasting blood glucose level. Using ADA criteria only has been found to underestimate the prevalence of diabetes and misses those individuals with impaired glucose tolerance (IGT), a pre-diabetic state [4]. The disadvantage of both these diagnostic approaches is that they require the patient to fast and if need confirming, would require a second fasting sample. Glucose also has a large biological and diurnal variation and depends on recent carbohydrate intake and the OGTT is fairly invasive [5]. In 2010, the ADA updated their diagnostic criteria to include an OGTT as well [6].

On the other hand, HbA1c, which is formed by the attachment of glucose to various amino groups of haemoglobin and has been used since 1977 for the long-term (2–3 month) glycaemic control follow up of diabetes, has recently been advocated by the ADA as a diagnostic tool. In 2009, the International Expert Committee of the ADA issued a statement proposing an HbA1c value of 6.5% (48 mmol/mol) as a diagnostic level for the diagnosis of diabetes. This value was chosen, as it was found to be the value after which the incidence of retinopathy, a common complication that often is present before the actual diagnosis of diabetes is made, is increased [7]. This test would be advantageous, as it does not require a fasting sample and has much less intraindividual variation.

The worldwide diabetes “epidemic” is expected to affect developing countries more than developed ones. Studies to determine the usefulness of HbA1c as a diagnostic tool in these populations are needed, as there is a paucity of data from these communities compared to Western countries. As there appear to be racial differences in HbA1c levels [8], [9], the purpose of this study was to assess the utility of an HbA1c value of 6.5% as a diagnostic tool for diabetes in our local Coloured (mixed ancestry) population, and to establish an optimum cut-off for this population.

## Results

A total of 946 subjects participated, comprising 642 random subjects between the ages 35–65 years and 304 voluntary subjects, age range 16–95. One hundred and twenty two subjects with known diabetes and 5 that did not consent for blood collection were excluded. Therefore, for this study, 819 subjects with the median (confidence interval) age of 52 (52, 54) were eligible.

Table 1 shows the general characteristics of the participants for this study. Although there were more females (722) than males (224) in the cohort, this is not reflective of the female to male ratio of the population, but of the willingness to participate. The females had significantly higher BMI and subsequently a higher prevalence of diabetes mellitus. No significant differences were observed between male and female HbA1c values; these ranged from 4.4% (25 mmol/mol) to 13.3% (122 mmol/mol) (median 5.8% (40 mmol/mol) and 5.9% (41 mmol/mol) respectively).

**Table 1:** Characteristics of all participants (946), stratified by gender.

Characteristics	Male (N = 224)	Female (N = 722)	p
Age (years)	57 (44, 68)	53 (43, 64)	0.023
BMI (kg/m^2^)	25.1 (21.9, 29.2)	30.3 (25.8, 34.8)	<0.0001
FBG (mmol/L)	5.5 (5.0, 6.5)	5.6 (5.0, 6.4)	0.567
PostBG (mmol/L)	6.4 (5.3, 8.4)	6.9 (5.7, 8.9)	0.013
HbA1c (%)	5.9 (5.5, 6.3)	5.8 (5.5, 6.3)	0.856

**Figure 1:**
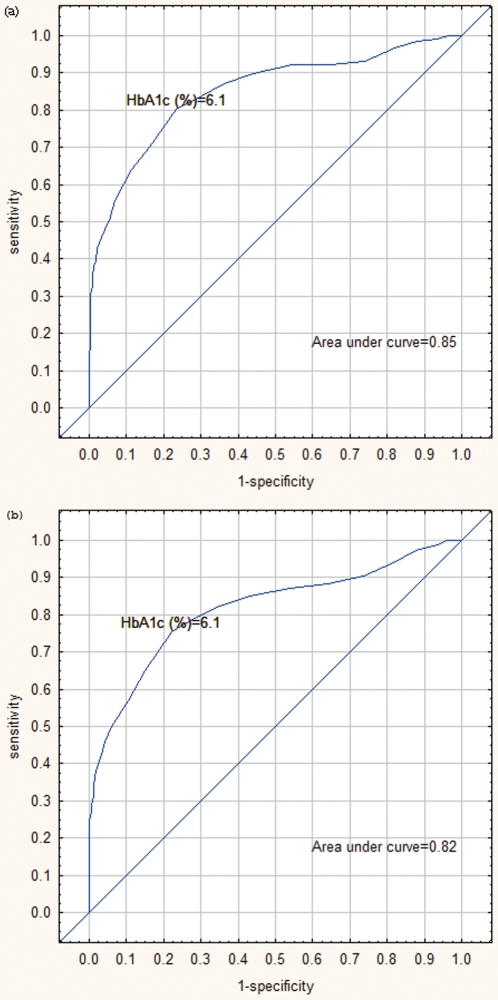
ROC curves depicting an HbA1c cut-off value of 6.1% as optimal for the diagnosis of DM according to fasting blood glucose and the OGTT. Figure 1a area under curve = 0.85, sensitivity = 80%, and specificity = 77%. Figure 1b area under curve = 0.82, sensitivity = 75%, and specificity = 78%.

FBG, fasting blood glucose; PostBG, post 2-hour blood glucose;

One hundred and seventeen subjects were newly diagnosed with diabetes using fasting blood glucose and 147 when using an OGTT. Using ROC curves, the optimal cut-off for HbA1c for diabetes as diagnosed according to either fasting blood glucose or OGTT gave a value of 6.1% (43 mmol/mol), AUC 0.85 and 0.82 respectively. The sensitivities at this cut-off were 80% and 75% respectively and the specificities 77% and 78% respectively (Figure 1). The ROC's were repeated with age categories of <60 and ≥60 years and the optimal cut-off for HbA1c as a screening tool for diabetes remained at 6.1% (43 mmol/mol) for both age categories.

Table 2 summarises the stratification of diabetic and normal subjects according to HbA1c cut-off of 6.5% (48 mmol/mol) and 6.1% (43 mmol/mol). When using a cut-off of 6.5% as recommended by the ADA, high specificities were obtained, but sensitivity was sacrificed. An HbA1c value of 6.5% (48 mmol/mol) gave a sensitivity of 50% and specificity of 95% using fasting blood glucose for diagnosis and a sensitivity of 46% and specificity of 96% when using an OGTT.

**Table 2:** Stratification of diabetic and normal subjects according to HbA1c cut-off of 6.5% and 6.1%.

	HbA1c<6.5%	HbA1c<6.1%	HbA1c≥6.5%	HbA1c≥6.1%
**Diagnosis using FBG**				
Normal (%)	97.1	83.3	2.9	16.7
DM (%)	49.6	19.7	50.4	80.3
**Diagnosis using OGTT**				
Normal (%)	97.5	80.5	2.5	19.5
DM (%)	54.1	24.7	45.9	75.3

## Discussion

Type 2 diabetes is increasing in developing countries due to growth and aging of the population, and urbanisation with its introduction of an unhealthy diet and increasing obesity and sedentary lifestyles. In a recent article Bradshaw *et al* estimated that 5.5% of all South Africans ≥30 years had diabetes [10]. Levitt *et al* studied the prevalence of diabetes in 974 residents from the mixed ancestry (coloured) community of Mamre, a rural town near Cape Town in the Western Cape. The age standardized prevalence of type 2 diabetes in the age group 30–65 years, South African mixed ancestry population group was 10.8% and that of impaired glucose tolerance (IGT) 10.2% [11]. Age, physical inactivity, family history of diabetes and waist circumference was all identified as independent risk factors [11]. Another study that specifically examined the prevalence of diabetes in elderly coloured subjects found a prevalence of 28% for men and 29% for women [12], [13]. A population of Indian origin studied in sub-Saharan Africa found prevalences of diabetes of between 12 and 13%. This increasing incidence of diabetes with its subsequent complications in Sub-Saharan African countries such as South Africa places an even greater burden on health care systems already buckling under the challenges of diseases such as malaria, tuberculosis and HIV [14]. The incidence of diabetes in this study was more than 20% using either fasting blood glucose or an OGTT for diagnosis. For this reason, a quick and simple diagnostic test such as HbA1c would be advantageous.

HbA1c is formed by the attachment of glucose to various amino groups. The Diabetes Control and Complications Trial (DCCT), which determined HbA1c using a precise HPLC method, showed that a reduction of HbA1c led to a reduction in diabetic complications [15]. This opened the door for HbA1c standardization, as there are numerous analytical methods available for its determination [16]. In 2009, the International Expert Committee of the ADA issued a statement proposing an HbA1c value of 6.5% (48 mmol/mol) as a diagnostic level for the diagnosis of diabetes. This value was chosen, as it was found to be the value after which the incidence of retinopathy, a common complication that often is present before the actual diagnosis of diabetes is made, is increased [7]. Westgard expressed concern about using the HbA1c value as a diagnostic test, as even though the test has been “standardized”, CAP proficiency testing still shows significant biases between methods [17].

Previous studies proposed using HbA1c as a screening tool for the detection of diabetes, but their cut-off values differed [18]–[21]. In the present study, we have shown a cut-off of 6.1% (43 mmol/mol) as optimal for all mixed ancestry ages groups from South Africa. Our findings are similar to those reported by Bennett *et al* who performed a meta-analysis of nine studies and found that most recommended an HbA1c cut off of 6.1% (43 mmol/mol) as optimal [19]. A recent study by Kumar *et al* assessed the validity of HbA1c as a screening and diagnostic test for diabetes. They found a value of 6.1% (43 mmol/mol) had optimal sensitivity and could thus be used for screening and 6.5% to have optimal specificity and could thus be used as a diagnostic test [21]. In 2008, Saudek *et al* found an HbA1c cut off of 6.0% (42 mmol/mol) to be optimal for screening and 6.5% (48 mmol/mol) for diagnosis of diabetes [20]. Rowley *et al* found that a value of 7% (53 mmol/mol) had the best specificity and could therefore be used as a diagnostic test. However, this study was published in 2005 [18].

Our study has the following strengths: (1) the number of participants is large and from the same population group and (2) the diagnosis of diabetes was made using either fasting blood glucose or an OGTT and compared.

However, our study also has some limitations: (1) the haemoglobin and iron status was not determined simultaneously, and these can affect the red blood cell survival and thus render HbA1c levels unreliable; (2) the presence of renal impairment can also affect HbA1c levels and was not investigated; (3) HbA1c levels are affected by various haemoglobinopathies and thalassaemias and these were not determined; (4) the extent to which haemoglobin glycation occurs also varies between individuals and may be affected by environmental parameters, such as lipid peroxidation and hereditary factors, and (5) medication use, specifically those such as antiretrovirals which may affect glucose metabolism, was not examined Although we did not look for haemoglobinopathies and thalassaemias, their incidence is fortunately low in this population. Another possible shortcoming, but not necessary limitation, of this study was that HbA1c was not determined on an HPLC-based method as used in the DCCT and may thus be more prone to interferences. However, the assay is NGSP- certified and had acceptable CV's.

In conclusion, we recommend an HbA1c value of 6.1% (43 mmol/mol) as an optimal cut off to screen for diabetes in our local population. A cut off of 6.5% (48 mmol/mol) would be a good diagnostic tool with its high specificity (95% using fasting blood glucose and 96% using OGTT), however the low sensitivity limits the use of this value (50% using fasting blood glucose and 46% using OGTT).

Our study emphasizes the need for evidence based values to be established in various population groups.

## Methods

### Ethical Considerations

The study was approved by the Cape Peninsula University of Technology, Faculty of Health and Wellness Sciences Ethics committee and the University of Stellenbosch Ethics committee (N09/03/090). The study was conducted according to the Declaration of Helsinki. All participants signed written informed consent after all the procedures had been fully explained in the language of their choice. In addition, permission was also sought from other relevant authorities such as the city and community authorities. These authorities granted permission to operate in the community and also to make use of designated places such as community halls or nearby schools for data and samples collection. Patient confidentiality was maintained at all times.

### Research Setting

Bellville-South is located within the Northern suburbs of Cape Town, South Africa and is a traditionally a Coloured township formed in the late 1950s. In the South African context, the term township usually refers to the often underdeveloped urban living areas that, under the Apartheid regime, were reserved for non-whites. According to the 2001 population census, its population stands at approximately 26 758 with 80.48% (21 536) consisting of the Coloured (mixed race) [22]. The predominant language in this community is Afrikaans and most of the residents of this community have lived there for over five years while others have been there for their entire lives.

### Research Design and Study Population

This was a cross-sectional quantitative study aimed at establishing a cohort that can be followed up in randomly selected coloured subject. The data was collected mid January 2008 to March 2009. Using a map of Bellville South, multistage stratified random sampling was approached as follows: From a list of streets from each stratum, the streets were then classified as short, medium and long streets based on the number of houses. Streets with houses ≤22 were classified as short, medium; houses 23–40 and long streets were >40 houses. A total of 16 short streets representing approximately 190 houses, 15 medium streets representing approximately 410 houses and 12 long streets representing approximately 400 houses were randomly selected across the different strata. From the selected streets, all household members meeting the selection criteria were invited to participate in the study.

### Recruitment Strategy

Information regarding the project was disseminated to the local residents through the local radio station, community news paper, brochures and fliers; the latter bearing information about the project and distributed through school children and taxis to the local residents by the recruitment team. This team consisted of unemployed matriculants and was managed by a qualified retired nurse from the community. Recruited subjects were visited by the recruitment team the evening before participation and reminded of all the survey instructions. These included overnight fasting, abstinence from drinking alcohol or consumption of any fluids in the morning of participation. Furthermore, participants were encouraged to bring along their medical/clinic cards and/or medication they were currently using.

### Pre-participation counseling

All participants except the self reported diabetic subjects, confirmed by either medical card record or drugs in use, had blood taken for fasting blood glucose and underwent a 75 g oral glucose tolerance test (OGTT) as prescribed by the WHO. DM was diagnosed both according to previous ADA criteria using only a fasting blood glucose [3] and according to the WHO 2006 criteria [2] using a 75 g OGTT.

Blood samples were transported daily in an ice-pack box for processing at an accredited laboratory. Plasma glucose was measured by enzymatic hexokinase method (Cobas 6000, Roche Diagnostics). HbA1c was assessed by turbidimetric inhibition immunoassay (Cobas 6000, Roche Diagnostics). This method is National Glycohemoglobin Standardization Programme (NGSP) certified. The assay has a within-run CV of 1.4% and between run CV of 2.8%.

### Statistical Analysis

Statistical analysis of the data was performed using STATISTICA (STATISTICA 9, StatSoft Inc 1984–2009). The continuous variables are presented as median (confidence interval) or means ± standard deviation (SD), and categorical variable are expressed in percentage. For data where the normality assumptions were suspect, the Mann Whitney U test was used. Diabetes was diagnosed according to both WHO and ADA criteria; the ADA diagnostic cut-off of 6.5% for HbA1c was tested separately for each group. A Receiver operator characteristic (ROC) curve for both ADA and WHO diabetes criteria were generated and the area under the curve (AUC) calculated. Test sensitivity, specificity positive predictive value (ppv) and negative predictive value (npv) was calculated.
